# Genes Involved in DNA Damage Cell Pathways and Health of the Oldest-Old (85+)

**DOI:** 10.3390/genes14091806

**Published:** 2023-09-15

**Authors:** Maja Šetinc, Matea Zajc Petranović, Goran Slivšek, Sandra Mijač, Željka Celinščak, Anita Stojanović Marković, Vesna Bišof, Marijana Peričić Salihović, Tatjana Škarić-Jurić

**Affiliations:** 1 Institute for Anthropological Research, 10000 Zagreb, Croatia; maja.setinc@inantro.hr (M.Š.); zeljka.jurcic@gmail.com (Ž.C.); astojanovic@bfm.hr (A.S.M.); mpericic@inantro.hr (M.P.S.); tanja@inantro.hr (T.Š.-J.); 2 Faculty of Medicine, University of Zagreb, 10000 Zagreb, Croatia; goran.slivsek@xnet.hr (G.S.); sandra.mijac@vip.hr (S.M.); vbisof@gmail.com (V.B.); 3 Department of Science and Research, Children’s Hospital Srebrnjak, 10000 Zagreb, Croatia; 4 Faculty of Medicine, Josip Juraj Strossmayer University of Osijek, 31000 Osijek, Croatia

**Keywords:** oldest-old, ageing, self-rated health, functional ability, DNA damage repair, single nucleotide polymorphisms, *TERC*, *MRE11A*, CDKN2B, Croatia

## Abstract

Some sources report a connection of cellular senescence with chronic pathological conditions; however, the association between particular cellular processes and general health is rarely examined. This study aims to test the relationship of general health with DNA damage pathways that play a crucial role in senescence. The association of ten selected SNPs with subjective and objective general health and functional ability indicators has been tested in 314 oldest-old people from Croatia. Multivariate logistic regression was employed to simultaneously test the impact of variables potentially influencing targeted health and functional ability variables. The best model, explaining 37.1% of the variance, has six independent significant predictors of functional ability scores: rs16847897 in *TERC*, rs533984 in *MRE11A*, and rs4977756 in *CDKN2B*, chronic disease count, Mini-Mental State Examination scores, and age at surveying. In conclusion, the examined ten loci involved in DNA damage repair pathways showed a more significant association with self-rated health and functional ability than with the number of disease or prescribed medicaments. The more frequent, longevity-related homozygote (GG) in rs16847897 was associated with all three aspects of self-assessments—health, mobility, and independence—indicating that this *TERC* locus might have a true impact on the overall vitality of the oldest-old persons.

## 1. Introduction

Ageing is a physiological process defined by the constant loss of functions and stability at the molecular, cellular, tissue, and organ levels, which leads to the deterioration of the general state of health and physical fitness [1], changes in psychological and social functionality [2], and ultimately to death. The average life expectancy has increased compared to previous generations [3], which was mainly achieved due to better health care (both prevention and curation), hygiene, nutritional sufficiency, and reduced child mortality. In addition to the increase in average length of life, more and more people live to a very old age, earning the title of the oldest-old. The American Geriatric Society and the World Health Organisation define the oldest-old as those over 80, while the British Geriatrics Society uses the age of 85  years as the threshold. Projections show that the world’s oldest-old population is likely to triple from 2015 to 2050 [4].

Longevity is more complicated to explain than an expansion in the average lifespan; it is a multifactorial trait in which the main role is played by the interactions of genes and the environment, as well as the complex interplay of multiple genes and signalling pathways [5]. Processes that lead to ageing at the cellular level—i.e., cellular senescence—have been identified in various organisms, and the main detected traits of senescent cells and mechanisms of senescence induction include changes in genome stability [6], telomere shortening [7], disturbed proteostasis in response to oxidative damage [8], and epigenetic changes [9]. DNA damage (nucleotide and deoxyribose damage, formation of DNA-protein cross-links, DNA chain break) as a signal activates a series of biochemical reactions triggering a variety of cellular responses, the key being the recognition of the DNA damage signal and the initiation of downstream cascade signalling biochemical reactions [10]. This complex network of processes centred around DNA damage includes proteins that have an active role in the repair of damaged DNA, can modulate the activity of the DNA damage response, or act as controllers of checkpoints in cell cycle progression. Among them are, for example, proteins of the MRN complex (Mre11-Rad50-Nbs1, responsible for linking double-strand break repair with cell cycle checkpoint functions) [11], the interleukin-6 (IL6) protein (a cytokine that affects the DNA damage sensor histone variant γ-H2AX and therefore DNA repair) [12], and also the proteins of tumour suppressor genes such as *CDKN2A* and *TP53* [13]. Furthermore, critical shortening of telomeres is also registered as DNA damage, so proteins involved in telomere maintenance could also fall into this group. For example, the human telomerase RNA (hTR) component of telomerase encoded by *TERC* gene is capable of regulating the ATR-mediated DNA damage response and involved in the activation of the catalytic subunit of DNA-dependent protein kinase (DNA-PKcs) that phosphorylates heterogeneous nuclear ribonucleoprotein A1 (hnRNP A1), an RNA-binding protein, in a telomerase-independent manner [14,15]. DNA damage, if not successfully repaired, leads to tissue senescence, characterised by a decreased number of viable cells and the senescence-associated secretory phenotype (SASP). Two processes lead to this status: cellular death with the clearing up of dead cells and processes leading to cellular senescence. The failure of these mechanisms usually gives rise to the malignant transformation of damaged cells.

There are numerous examples directly connecting cellular senescence with some chronic pathological conditions [16,17,18,19]. However, the association between particular cellular processes and general vitality and health is not widely examined.

In addition to ageing at the cellular level, there are number of questions considering the measures for the overall health of the oldest-old. Namely, it is still a matter of consideration what is the best measure of the quality of life in old age; e.g., whether it is the low number of chronic conditions or the overall feeling of well-being and functional ability. It has been widely recognised that the dynamics of the ageing process are not the same in all individuals and that genetic and environmental (lifestyle) differences substantially impact the speed and modalities of this process. Among middle- and old-age peers, there are individuals who better tolerate stress, suffer from fewer diseases, take fewer medications, etc. The situation is even more complex; namely, there is an increasing body of evidence that the number of chronic pathological conditions is not the best predictor of mortality and that lesser scores of those objective measures are not the most proximate indicator of overall vitality and a guarantor for longevity.

The World Health Organisation defines healthy ageing as the development and maintenance of functional abilities that promote well-being in the lives of older adults [20]. The importance of considering a person’s functional status has been widely recognised for some time. Namely, functional status has been shown to predict mortality and some morbidities reliably [21]. Functional ability, or capacity, is an assessment of a person’s ability to perform daily activities without the assistance of others [22]. This assessment incorporates several factors, including the level of mobility and independence. In general, a person’s mobility means being able to move around without assistance, while a person’s independence implies the capability of doing tasks without someone else’s help [23].

Besides assessing functional status, it has been demonstrated that self-rated health is also crucial in determining a person’s health outcome [24]. Self-rated health is widely considered a reliable measure for determining mortality and morbidity because it can very accurately predict them [25]. Wuorela et al. have proved that self-rated health is almost as good a predictor of mortality risk in older adults in a clinical setting as objective health status [26]. From all this, it is clear that functional status and self-assessed health play an important role in determining a person’s overall vitality.

This pilot study’s goal was to provide some answers to the question of whether overall vitality and health can be related to cellular senescence processes, in which DNA damage cell pathways play a principal role. Having all the considerations listed above in mind, the specific aim of the present study was to investigate the relationships of the ten selected SNPs in genes involved in DNA damage response cellular pathways with subjective and objective indicators of general health in the oldest-old residents of retirement homes in Zagreb, Croatia.

## 2. Materials and Methods

### 2.1. Study and Participants

The study was conducted as a part of the interdisciplinary project “HEalth, CUltural, and Biological determinants of longevity: Anthropological perspective on survival in very old age” (acronym: HECUBA) that comprehensively investigated the relationship between numerous biological and health-related features in long-lived individuals.

A field study was carried out from 2007 to 2009, and the participants were recruited from 13 retirement homes in Zagreb, Croatia. All residents of these homes aged 85 years and older were given information about the research procedure and were invited to participate voluntarily, with age being the sole inclusion criterion. Extensive face-to-face interviews were conducted by trained interviewers as part of this field study, and blood samples were acquired for genetic analyses. The initial sample consisted of 345 oldest-old persons, but due to incomplete health status information or genotyping failure, the final sample for the present study amounts to 314 participants.

### 2.2. Selection of Genetic Loci and Genotyping Method

Forty-three candidate genes for longevity were initially chosen for the HECUBA study from publicly available literature databases (PubMed as well as repositories specialised for human longevity, i.e., https://genomics.senescence.info/longevity/, http://ageing-map.org/, accessed on 25 May 2023) on the grounds of their strong or repeatedly reported association with human longevity and involvement in various metabolic pathways (insulin/IGF signalling, DNA repair, lipid metabolism and transport, pro/antioxidant processes etc.). From this wider set of candidate genes, ten genes were selected for the present study based on the criteria of their involvement in DNA damage cell repair pathways, and their involvement and interrelations were confirmed by using the free software STRING, https://string-db.org/ (accessed on 2 June 2023). In each candidate gene, one single nucleotide polymorphism (SNP) was selected, the one for which the most statistically significant association with longevity was found by reviewing the literature databases. Table 1 contains information about the selected SNPs: rsID, chromosome position, nearest gene, alleles, minor allele frequencies (MAF), genotyping success rate, and Hardy–Weinberg equilibrium (HWE) *p*-values, while the relationship between the 10 selected genes and the particular cellular pathways is presented in the Supplementary Materials.

The genomic DNA was isolated from the peripheral blood using the salting-out method [27]. Genotyping was conducted in a commercial laboratory using the Kompetitive Allele-Specific PCR (KASP) method. The KASP genotyping assay is a form of competitive allele-specific PCR combined with a homogeneous fluorescent SNP genotyping system, determining the alleles at a specific locus within genomic DNA [28].

### 2.3. Questionnaire

The questionnaire used in the survey was adjusted to the specific population of oldest-old persons and consisted of six semi-structured sections (for details, see Perinić Lewis et al. [29]). However, only a few variables from the health section addressing current general health status were used for the present analysis.

The objective general health status is represented by three variables: (1) the number of current chronic diseases and conditions, (2) the number of medications taken per day, and (3) the Mini-Mental State Examination test scores (MMSE), which is a standardised scale that assesses general cognitive functioning and measures eventual cognitive impairment.

The subjective general health status is represented by four variables that contain scales of self-assessment of current health and functional ability. Participants rated their general health, mobility, and independence on a five-point response scale (from 1 = poor to 5 = excellent). They also compared their subjective health with that of their peers on a four-point response scale (from 1 = worse than to 4 = better than). The scores of those four self-rated health and functional ability variables were joined into various combinations: all four variables together, three variables together (all four except the self-rated health in comparison with others), and variables that combine two types of self-perception: health (self-perceived health and health in comparison with others) and functional ability (mobility and independence).

### 2.4. Data Analysis

All the above-described self-rated health and functional ability variables were binarised using different cut-offs, and their categories were compared with the genotype distribution of the ten selected SNPs using the chi2-test. The differences in the values of the objective health status variables as well as those of summed self-rated health and functional ability scores across genotypes in ten genetic loci, were tested using analysis of variance (ANOVA) and post hoc test. Statistical significance was set at *p* < 0.05 in all cases.

The general health variable showing the most significant association with a genetic locus was selected as the dependent variable in the multivariate logistic regression model. This analysis was used as a means for simultaneously testing the impact of the health-associated SNP with other variables that could have a potential influence on the binary dependent variable that showed the most significant association with those particular SNPs.

All statistical analyses were performed using the SPSS software package 21.0 [30].

## 3. Results

The significant relations of the ten SNPs with three objective indicators of general health as well as with subjective self-rated health and functional ability scores are presented in Table 2 for quantitative and in Table 3 for binarised health variables.

As presented in Table 2, out of ten examined SNPs, three (in the genes *TERC*, *MRE11A*, and *CDKN2B*) were significantly associated (*p* < 0.05) with subjective health and functional ability variables, and in one case with MMSE score. On the other hand, three different SNPs (in the genes *TXNRD1*, *IL6*, and *PARK7*) were related to objective general health variables: the number of diseases and the number of medicaments consumed per day.

### 3.1. Subjective General Health

According to the ANOVA results, the most significant relations were obtained between rs16847897 in the *TERC* gene (ANOVA *p* range: 0.001–0.006) and three combinations of self-rated health and functional ability scores. a post hoc test showed that in all cases, the more common GG genotype was associated with higher scores. Also, persons with this genotype had significantly higher mean MMSE values, indicating preserved cognitive function (ANOVA *p* = 0.014). A significant association with the same set of health variables was also obtained for rs533984 in the *MRE11A* gene (*p* ranging from 0.005 to 0.029) and for rs4977756 in the *CDKN2B* gene, with higher (better) scores obtained for the less frequent genotypes in both genes. It should be emphasised that in previous research, GG was assigned as a longevity genotype in rs16847897 [31,32] and in rs4977756 [33], which makes this result of the subjective health and functional ability assessment consistent with the expected longevity of these individuals. However, for rs533984 in the *MRE11A* gene, better scores were associated with the AA genotype, which was not previously related to longevity [34,35].

Complementary results were seen when the chi2-test was performed using binarised health variables (presented in Table 3). The same set of loci was significantly and in the same direction associated with self-rated health and functional ability status variables. The most significant relationship (*p* = 0.00026) was found between rs16847897 in the *TERC* gene and the sum of self-rated mobility and independence scores (with 7+ as the cut-off value for a better outcome). It should be stressed that this *TERC* gene SNP is associated with all three aspects of self-assessment: health, mobility, and independence, and additionally with the Mini-Mental State Examination scores. On the other hand, the investigated *MRE11A* locus (rs533984) showed a relation only with functional ability scores (with the AA genotype being associated with better scores) but not with self-rated health. Additionally, a significant relation was found between the lower number of drugs taken daily and the less frequent AA genotype of this locus. In a set of subjective general health variables in their binarised version, the only additional significant locus was rs1333049 in the *CDKN2A* gene, showing an association of the more frequent and longevity-related GG genotype with better self-rated mobility scores.

### 3.2. Objective General Health

When objective general health quantitative variables were considered (Table 2), a larger number of diseases and/or a greater number of drugs taken daily were associated with three SNPs: rs17202060 in the *TXNRD1* gene, rs1800795 in the *IL6* gene, and rs225119 in the *PARK7* gene. In qualitative analysis (Table 3), rs225119 in the *PARK7* gene showed an association with gender-specific medians of the number of diseases (with a higher number of diseases being related to a less frequent, longevity-related TT genotype). rs17202060 in the *TXNRD1* gene showed the association of TC heterozygotes with a larger number of chronic diseases and/or conditions (4+). Here, a new locus—rs2706372 in the *RAD50* gene—showed the association of CT heterozygotes with fewer chronic diseases (below gender-specific medians).

### 3.3. Multivariate Analysis

The sum of self-rated mobility and independence scores with a cut-off value of 7+ was the general health variable most significantly associated with a genetic locus. Therefore, this binary self-rated general health variable was used as the dependent variable in multivariate logistic regression analysis. This analysis was performed to investigate whether the significant relation obtained here with rs16847897 in the *TERC* gene would remain when other loci (which showed some association with self-assessed health and functional ability) were included in the model. The model included the following independent variables: gender, age at measurement, MMSE score, number of chronic diseases and/or conditions, and number of drugs taken daily, as well as the four genetic loci (*TERC*, *MRE11A*, *CDKN2A*, and *CDKN2B*).

The resulting model (Table 4), explaining 37.1% of the variance, has nine independent variables, and six of them presented themselves as the independent significant predictors of functional ability scores in the oldest-old Croatian sample.

Among non-genetic variables, the lower number of chronic diseases (OR = 0.738, 95%CI 0.645–0.844, *p* < 0.001) and higher MMSE score (OR = 1.125, 95%CI 1.053–1.203, *p* = 0.001), as well as lower age at measurement (OR = 0.974, 95%CI 0.951–0.996, *p* = 0.024) were significant predictors of preserved functional ability scores. Within the context of other variables, gender and number of daily used medicaments were not shown to be significantly related to the target functional ability variable.

Considering genetic loci, out of four tested SNPs, three were significantly related with the dependent functional ability variable: AA genotype at the rs533984 in *MRE11A* gene (OR = 2.536, 95%CI 1.055–6.094, *p* = 0.037), GG genotype at the rs4977756 in *CDKN2B* gene (OR = 2.966, 95%CI 1.051–8.369, *p* = 0.040), and GG genotype at the rs16847897 in *TERC* gene (OR = 2.648, 95%CI 0.903–7.763, *p* = 0.076) increase the chance for a better functional ability score. Although the impact of GG homozygotes when contrasted with joined CC and GC genotypes in the *TERC* locus reached only a 10% significance level, it should be noted that the overall impact of the rs16847897 locus within the presented model was highly significant (*p* = 0.001) when all three genotypes were contrasted.

## 4. Discussion

Functional status and self-rated health play an important role in determining a person’s overall vitality and are considered a reliable measure for determining mortality and morbidity. Despite numerous examples directly linking cellular senescence to some chronic pathological conditions, the association between DNA damage leading to cellular senescence and the general vitality and health of a person has not been widely investigated. In our pilot study of the association of 10 SNPs in genes involved in the DNA damage signalling pathway with self-assessed subjective and objective general health in 85-year-olds and older, the role of three SNPs was highlighted univariately and multivariately. Specifically, rs16847897 in the *TERC* gene, rs533984 in the *MRE11A* gene, and rs4977756 in the *CDKN2B* gene show independent associations with functional ability scores.

The *TERC* gene, located on chromosome 3q26, encodes the RNA component of telomerase (hTR), a ribonucleoprotein that serves as a template necessary for the elongation of telomeric DNA [36,37]. Since telomeres shorten with each cell division until they reach a critically short length, they act as a molecular clock that controls the cells’ replicative potential [38] and entry into senescence [39]. Telomere attrition is thus considered one of the hallmarks of ageing [40] and has been the focus of ageing research. Leukocyte telomere length (TL) has been recommended as a measure of biological ageing since it is commonly observed to be shorter in individuals with age-related conditions such as Alzheimer’s disease and vascular dementia [41,42]. While TL differs between tissues, the length of telomeres in leukocytes correlates with TL in most other tissues and can serve as a proxy for many tissue types [43]. Leukocyte TL has been linked to the intronic SNP rs16847897 downstream of the *TERC* gene in large UK cohorts [44] and the Chinese Han population [31]. The C allele of rs16847897 was associated with a shorter mean telomere length in the Chinese Han population research, which corresponded to an average age-related telomere attrition of about four years. In the Southern Italian population, the C allele of rs16847897 enhanced the chance of earlier development of Alzheimer’s disease [45] but did not significantly correlate with human lifespan in a follow-up study [46]. In a previous study on the same Croatian sample of the oldest-old, we found that the other allele, G, contributed to longevity by increasing the probability of reaching the longevity threshold age of 90 years [32]. The G longevity allele is a major allele in each of the European populations mentioned above and a minor allele in the Chinese Han population. The G allele of *TERC* rs16847897 was significantly associated with better subjective health, functional ability, and MMSE score in our 85+ sample, and the multiple logistic regression model showed that the relationship of this SNP with functional ability remained significant even when another eight variables were simultaneously tested.

Expression of telomerase is shut off during embryonic differentiation, and the coding genes remain unexpressed in most human somatic cells, except in male germ cells, activated lymphocytes, and certain types of stem cell populations [47], with telomerase reverse transcriptase gene (*TERT*) expression being a major regulator and limiting factor of telomerase activity [48]. The expression of the telomerase RNA component in adult tissues has been debated, with some reporting a ubiquitous expression in somatic cells [49] and others finding an expression pattern correlating with telomerase activity and limiting it mostly to dividing cells in testis, lymphoid follicles, and regenerative epithelial cells, but with some exceptions [48,50]. SNP rs16847897 in the *TERC* gene might, therefore, in later stages of life, have a telomerase-dependent effect in those cells where the telomerase gene is still being expressed. It could enhance telomerase activity in stem cells, increase their proliferative capability, or have a similar beneficial effect on clonal expansion of lymphocytes in peripheral lymphoid organs [51], enabling a swifter and stronger immune response. The better replicative potential of the cells aids in the maintenance of tissues and slows the accumulation of senescent cells, which could, in turn, result in lesser biological age and healthier ageing overall, including better functional and cognitive status and self-rated health.

However, *TERC* rs16847897 could also have a telomerase-independent effect on cell cycle progression via the interaction between the hTR and the checkpoint kinase ATR (Ataxia Telangiectasia and Rad3-related protein), an essential kinase that initiates cell cycle arrest in case of a DNA lesion that is causing the replication fork stalling [52]. Kedde et al. (2006) found that ectopic expression of hTR from the *TERC* gene inhibits ATR independently of telomerase activity and telomere length, causing defects in ATR-dependent checkpoints [13], while inhibiting the expression of *TERC* caused a cell cycle arrest through the p53/CHK1-dependent pathway even in the absence of apparent DNA damage. Additionally, hTR was reported to potentially stimulate phosphorylation of hnRNP A1 [14], a protein with many RNA-related functions that was also shown to play a role in telomere length maintenance [53], by DNA-PK, thus affecting its nucleic acid binding properties. Since DNA-PK interacts with and is targeted by ATR in response to UV exposure, hTR might be the link between the two—a signalling molecule that acts in response to UV-induced DNA damage [14].

Besides rs16847897, the multiple logistic regression model showed that another two genetic loci were also independently associated with functional ability in our 85+ sample: rs533984 in the *MRE11A* gene and rs4977756 in the *CDKN2B* gene, together with the age at measurement, MMSE score, and number of chronic diseases and conditions. The *MRE11* gene is part of the MRN complex, which consists of the proteins meiotic recombination 11 homolog A (MRE11A; also known as MRE11), RAD50, and Nijmegen breakage syndrome 1 (NBS1). With a vital role in both homologous recombination and non-homologous end-joining, the central component of the complex, MRE11A, shows activity as a single-stranded (ss)DNA endonuclease and as a double-stranded-specific 3′-5′ exonuclease. It is crucial for the preservation of telomere integrity, double-strand break repair, recombination, and meiosis [11,54,55,56,57], but has also been implicated in protecting mtDNA from oxidation, cytoplasmic leakage, and failure of ATP production [58]. Dato et al. discovered that the intronic *MRE11A* rs533984 G allele was strongly linked with extreme survival in women. Additionally, interactions between this SNP in the DNA-repair pathway and SNPs from the insulin/insulin-like growth factor signalling pathway (IIS) were significantly associated with longevity, as demonstrated by the two- and three-loci interaction analysis [34]. In our Croatian sample [35], out of 43 longevity genes’ loci, rs533984 was the only one that differentiated old (85+) and young (20–35 yrs) cohorts, with the G allele being more frequent in the former group. Despite those two findings pointing to the G allele as associated with longevity phenotype, the results presented here relate minor A allele with better functional ability status in the oldest-old cohort.

Intronic genetic variation rs4977756 is located in the 9p21.3 chromosome region that has been strongly associated with an increased risk for coronary heart disease [59,60], with the risk variants comprising a single haplotype that spans approximately 58 kb [60]. It is a highly complex genomic region that includes the tumour-suppressor genes *CDKN2A* and *CDKN2B. CDKN2A* gene encodes splice variants p16^INK4A^ and p14^ARF^, and *CDKN2B* encodes p15^INK4B^. The proteins p16^INK4A^ and p15^INK4B^ are cyclin-dependent kinase (CDK) inhibitors, inhibiting retinoblastoma phosphorylation by CDK4/6, and play a role in cell cycle regulation and senescence [61]. The locus also encodes CDKN2B-AS1, also known as ANRIL, a long non-coding RNA spanning over 126 kb and overlapping the *CDKN2B* gene [62] that has been shown to be aberrantly expressed in various tumours and diseases [61]. ANRIL also has a pivotal role in the regulating of *CDKN2A/B* expression through a cis-acting mechanism, thus serving as a regulator of proliferation and senescence [63,64]. SNPs in this region have been associated with longevity in various studies [33,65,66,67]. The G allele of rs4977756, which is enriched in centenarians, was shown to be protective against coronary artery disease and diabetes and was associated with the parent’s age at death [33,66]. That same allele was associated with higher self-rated health and functional ability scores in our oldest-old sample. rs1333049, located in the *CDKN2A* region of the 9p21.3, on the other hand, was significantly associated only with self-rated mobility.

Out of the ten investigated SNPs with some involvement in the DNA damage pathway, four SNPs, namely in the genes *TXNRD1*, *IL6*, *PARK7* and *RAD50*, showed an association with some of the objective general health variables that were tested. CC genotypes of both rs17202060 in the *TXNRD1* gene and rs1800795 in the *IL6* gene and TT genotype of the *PARK7* gene rs225119 were associated with a larger number of diseases and a greater number of medicaments taken daily. The *TXNRD1* gene encodes thioredoxin reductase (TrxR), which plays a key role in maintaining redox homeostasis by reducing thioredoxins and other substrates. rs17202060 in this gene has been implicated in longevity in a study by Dato et al. that looked for associations of SNP–SNP interactions with longevity [34] and has been found to interact with dietary oxidative balance score to alter breast cancer risk [68]. Since free radicals damage DNA and can compromise genomic integrity, the involvement of this gene in longevity and cancer risk is not surprising. The *IL6* gene, on the other hand, encodes a member of the interleukin family that functions as both an inflammatory cytokine and an anti-inflammatory myokine and plays a critical role in immune defence [69,70]. It is also associated with insulin resistance and type 2 diabetes mellitus [71]. The CC genotype of rs1800795 has been associated with longevity in Italian male centenarians and the Turkish population [72,73], but the GG genotype has been associated with better survival in Swedish females [74]. Considering its dual pro-inflammatory and anti-inflammatory functions and the difference in allele frequencies between Caucasian subpopulations, it is possible that the effect of a certain allele differs between populations [75]. The *PARK7* rs225119 is located in a gene encoding a redox-sensitive chaperone protein that helps maintain mitochondrial homeostasis and protects neurons from oxidative stress, mutations of which have been found in patients with autosomal recessive early-onset Parkinson’s disease [76]. PARK7 also positively regulates androgen receptor-dependent transcription [77]. The minor allele T of *PARK7* rs225119 showed a significant association with a higher number of daily medicaments, as well as an association with the number of diseases, a variable that was binarised around median numbers that were gender-specific. Dato et al. listed allele G of rs225119 to be the one contributing to longevity (between alleles G and A) [34], which would be equivalent to allele C in our data (coded as alleles C and T), meaning that the allele related to longevity contributes to better overall health and fewer diseases. The CT heterozygote of rs2706372 in the *RAD50*, the gene for the ATPase component of the MRN complex, was also associated with fewer chronic diseases, which is partially in line with the finding of Flachsbart et al. that determined rs2706372 as a novel candidate with association for longevity [78].

Numerous studies have found a clear link between a person’s functional status and overall health, with a decline in functional abilities being associated with an adverse health outcome [79]. Decreased functional ability not only reduces the quality of life but can also lead to a higher burden of comorbidities, greater dependence on caregivers, institutionalisation and higher mortality rates [80]. Castellanos-Perilla et al. noticed that the incidence of functional status decline was associated with the number of comorbidities [81]. Also, according to Mutz et al., self-rated health assessment is crucial for collecting important health-related data and should be widely used [82]. Similarly, Latham et al. have pointed out that self-rated health is as good for predicting morbidity as it is for predicting mortality, as it significantly predicts disease [83]. This is also in line with a study by Ishizaki et al., which highlighted that self-rated health was independently and significantly associated with multimorbidity [84]. Therefore, it is possible to conclude that functional ability ranking and even a more subjective measure of a person’s health, such as self-rated health, could find their place in biomedical research as indicators of general health.

## 5. Conclusions

The overall picture of the obtained results from this preliminary study points to the following general conclusions:

- The examined ten genetic loci involved in the cellular pathways of DNA damage repair showed a more significant association with the self-rated health and functional ability scores than with the number of diagnosed diseases or prescribed drugs.
- Subjective and objective indicators of general health were almost entirely related to different genetic loci. Also, general cognitive function, as assessed by the MMSE score, was matched with subjective general health variables.
- rs16847897 in the *TERC* gene showed the most significant relationships with both quantitative and qualitative subjective general health variables and was associated with all three aspects of self-assessments: health, mobility, and independence (as well as with the MMSE score). The more frequent GG homozygote of this genetic locus, which has been associated with longevity in previous studies, was consistently related to better scores.
- The most significant relationship found between the binarised variable of functional ability (sum of self-rated mobility and independence) and rs16847897 in the *TERC* gene remained when an additional eight variables were included in the multivariate logistic regression model, indicating that the *TERC* locus investigated here might have a true impact on the overall vitality of the oldest-old people.
- For a firm conclusion on the connection of vitality and health with cellular ageing, a much more extensive experimental design is needed (more SNP loci such as in GWAS), and the results need to be confirmed in other populations.

## Figures and Tables

**Table 1:** General information about the selected ten genetic loci: rsID, nearest gene, chromosome position in GRCh38, as well as alleles (major/minor), minor allele frequencies (MAF), genotyping success rate, Hardy–Weinberg equilibrium (HWE) in the studied Croatian population.

SNP	Closest Gene	Chromosome Position (GRCh38)	Alleles (Major/Minor)	MAF	Genotyping Success Rate	HWE *p*-Value with Yates’s Correction *
rs225119	*PARK7*	1:7984301	C/T	0.425	0.979	0.815
rs16847897	*TERC*	3:169850328	G/C	0.292	0.979	0.996
rs2706372	*RAD50/IL13* region	5:132599785	C/T	0.272	0.960	0.997
rs1800795	*IL6*	7:22727026	G/C	0.448	0.948	0.562
rs4977756	*CDKN2B/ANRIL*	9:22068653	A/G	0.399	0.966	0.959
rs1333049	*CDKN2A*	9:22125504	G/C	0.470	0.972	0.319
rs533984	*MRE11A*	11:94466106	G/A	0.396	0.960	0.589
rs17202060	*TXNRD1*	12:104337068	C/T	0.336	0.960	0.812
rs1042522	*TP53*	17:7676154	C/G	0.238	0.976	0.448
rs50871	*ERCC2*	19:45359257	A/C	0.460	0.966	0.902

* Yates’s correction for continuity was applied for cases where the observed number of individuals in one of the cells of the Punnet square was smaller than 5.

**Table 2:** Significant relations of tested general health variables and ten genetic loci in the Croatian oldest-old sample: the results of the ANOVA and post hoc test.

Gene (rsID)	*p*-Value ANOVA	Variables	Means in More Frequent Homozygotes	Means in Heterozygotes	Means in Less Frequent Homozygotes	Genotypes with Significant Differences	*p*-Value Post Hoc Test
*TERC* (rs16847897)	0.001	Sum: self-rated health AND mobility AND independence	9.18	8.02	7.44	GG:CC GG:GC	0.005 0.001
	0.005	Sum: self-rated mobility AND independence	6.35	5.59	5.30	GG:CC GG:GC	0.022 0.005
	0.005	Sum: self-rated health AND self-rated health compared to that of their age peers AND mobility AND independence	11.81	10.63	10.08	GG:CC GG:GC	0.018 0.006
	0.006	Sum: self-rated health AND self-rated health compared to that of their age peers	5.48	4.97	4.72	GG:CC GG:GC	0.019 0.007
	0.014	MMSE	23.34	21.61	23.74	GC:CC GC:GG	0.060 0.007
*MRE11A* (rs533984)	0.005	Sum: self-rated mobility AND independence	6.04	5.60	6.84	AA:GA AA:GG	0.001 0.044
	0.016	Sum: self-rated health AND mobility AND independence	8.52	8.21	9.72	AA:GA AA:GG	0.004 0.028
	0.029	Sum: self-rated health AND self-rated health compared to that of their age peers AND mobility AND independence	11.13	10.82	12.45	AA:GA AA:GG	0.008 0.038
*CDKN2B* (rs4977756)	0.032	Sum: self-rated health AND self-rated health compared to that of their age peers AND mobility AND independence	10.60	11.28	12.22	GG:AA	0.010
	0.046	Sum: self-rated health AND mobility AND independence	8.11	8.58	9.43	GG:AA	0.014
*TXNRD1* (rs17202060)	0.024	Number of drugs per day	2.14	2.24	1.79	TT:CC TT:TC	0.033 0.006
	0.038	Number of diseases	6.14	5.49	5.24	CC:TC CC:TT	0.030 0.046
*IL6* (rs1800795)	0.028	Number of drugs per day	1.95	2.21	2.28	GG:CC GG:GC	0.018 0.025
*PARK7* (rs225119)	0.030	Number of diseases	5.65	5.53	6.54	TT:CC TT:TC	0.033 0.009

**Table 3:** Significant relations of dichotomised general health variables and ten genetic loci in the Croatian oldest-old sample: the results of the chi2-test.

Gene (rsID)	Variables	Class Where the Genotype(s) Is More Frequent	More Frequent Genotype(s)	Minor Allele	*p*-Value
*TERC* (rs16847897)	Sum: self-rated mobility AND independence	7–10	GG	C	0.00026
	Self-rated health	good, very good, excellent	GG	C	0.00133
	Sum: self-rated health AND mobility AND independence	7–15	GG	C	0.00148
	Sum: self-rated health AND self-rated health compared to that of their age peers	6–8	GG	C	0.004
	Self-rated mobility	good, very good, excellent	GG	C	0.005
	Sum: self-rated health AND self-rated health compared to that of their age peers AND mobility AND independence	10–18	GG	C	0.021
	Self-rated independence	good, very good, excellent	GG	C	0.029
	MMSE score	18+	GG, CC	C	0.010
*MRE11A* (rs533984)	Sum: self-rated mobility AND independence	7–10	AA	A	0.006
	Self-rated independence	very good, excellent	AA, GG	A	0.009
	Number of drugs per day/by median/	0–2 drugs per day	AA	A	0.005
*CDKN2B* (rs4977756)	Sum: self-rated health AND self-rated health compared to that of their age peers	6–8	GG	G	0.013
	Self-rated mobility	very good, excellent	GG	G	0.018
	Self-rated independence	very good, excellent	GG, GA	G	0.039
*CDKN2A* (rs1333049)	Self-rated mobility	very good, excellent	GG	C	0.031
*PARK7* (rs225119)	Number of diseases	Men: 6–12; Women: 7–12	TT	T	0.034
*RAD50* (rs2706372)	Number of diseases	Men: 6–12; Women: 7–12	CC, TT	T	0.026
*TXNRD1* (rs17202060)	Number of drugs per day	4+ (in both genders)	TC	T	0.050

**Table 4:** The multivariate logistic regression model for the dichotomised value of the sum of self-perceived mobility and independence in the Croatian oldest-old sample (N = 314). Independent variables are gender, age at measurement, mini-mental state examination (MMSE) score, number of chronic diseases and conditions, number of drugs used daily, and genotypic values of four genetic loci univariately related to the functional ability variables. The *p*-values of SNPs that passed the significance threshold of *p* < 0.05 are highlighted in bold.

Variables	*p*-Value	OR	95%CI
Gender (women are referent)	0.399	1.319	0.694–2.507
**Age at measurement (years)**	**0.024**	**0.974**	**0.951–0.996**
**MMSE score**	**0.001**	**1.125**	**1.053–1.203**
**Number of chronic diseases and conditions**	**0.000**	**0.738**	**0.645–0.844**
Number of drugs per day	0.682	0.974	0.859–1.105
***TERC* (rs16847897)**	**0.001**		
CC, GG are referent (vs. GC)	0.851	0.900	0.300–2.702
CC, GC are referent (vs. GG)	0.076	2.648	0.903–7.763
***MRE11A* (rs533984)**	**0.002**		
**GA, GG are referent (vs. AA)**	**0.037**	**2.536**	**1.055–6.094**
AA, GG are referent (vs. GA)	0.066	0.566	0.309–1.038
***CDKN2B* (rs4977756)**	0.063		
AA, GG are referent (vs. GA)	0.994	1.002	0.502–2.003
**AA, GA are referent (vs. GG)**	**0.040**	**2.966**	**1.051–8.369**
*CDKN2A* (rs1333049)	0.439		1.051–8.369
GC, GG are referent (vs. CC)	0.543	1.340	0.522–3.445
CC, GG are referent (vs. GC)	0.207	1.640	0.761–3.536

−2 Log Likelihood = 300.151; Percentage of correct classification = 74.205; Nagelkerke—R^2^ = 0.371.

## Data Availability

Genotypes of all participants are available in a fully anonymised dataset publicly available on the Zenodo repository (https://zenodo.org/record/7421684, accessed on 25 May 2023).

## Supplementary Materials

The following supporting information can be downloaded at: https://www.mdpi.com/article/10.3390/genes14091806/s1.
