# Supplementary material for: Genes Involved in DNA Damage Cell Pathways and Health of the Oldest-Old (85+)
Source: Genes (Basel). 2023 Sep 15;14(9):1806. doi: 10.3390/genes14091806 (PMC10530973; doi:10.3390/genes14091806)
Supplement: Supplementary file 1 [file genes-14-01806-s001.zip › genes-2550202-supplementary.pdf]

Supplementary document

The involvement of the 10 selected SNPs in DNA damage cell pathways, and their interrelations were checked and confirmed by using the free software STRING, https://string-db.org/ STRING is the knowledgebase and software tool for known and predicted protein-protein interactions. It includes direct (physical) and indirect (functional) associations derived from various sources, such as genomic context, high-throughput experiments, (conserved) co-expression and the literature.

List of protein names encoded by the 10 selected candidate genes was imported in the STRING, and then the software weighted and integrated the data and calculated confidence score for all protein interactions. The software built a network from 9 out of the 10 imported genes, and the statistical analysis showed that our network has significantly more protein-protein interactions than expected for a random set of proteins of the same size and degree distribution drawn from the genome (p=0.011). Such an enrichment indicates that the proteins are at least partially biologically connected, as a group. Graphically, their interrelation is as follows:

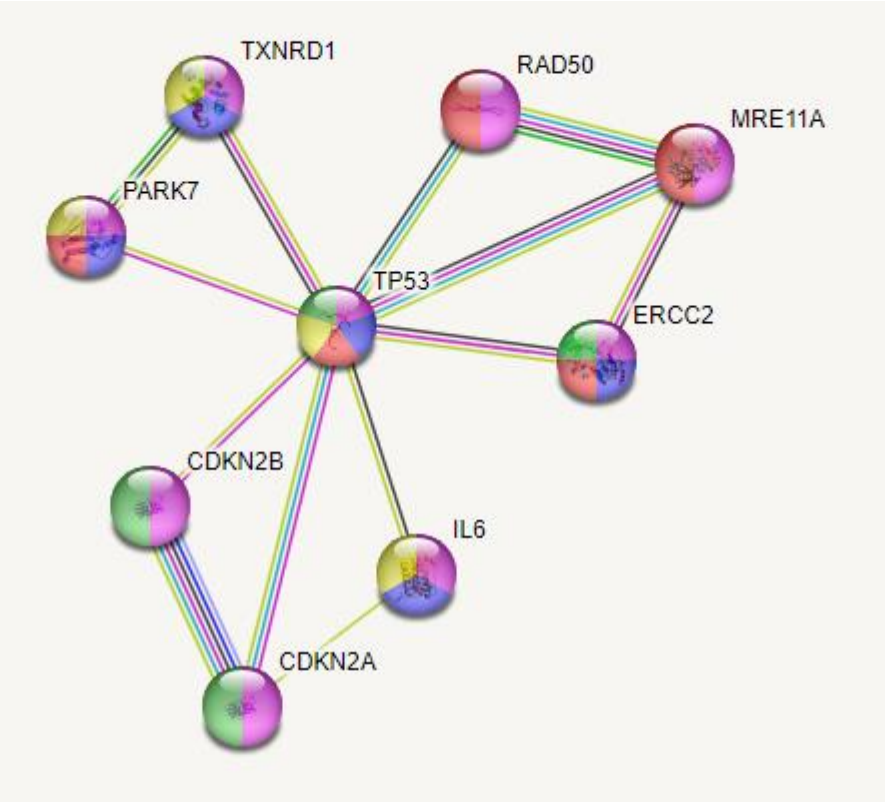

Network Stats

number of nodes:	9	expected number of edges:	6
number of edges:	13	PPI enrichment p-value:	0.011
average node degree:	2.89		
avg. local clustering coefficient:	0.835		

your network has significantly more interactions than expected (what does that mean?)

Functional enrichments in your network

explain columns

Biological Process (Gene Ontology)

GO-term	description	count in network	strength	false discovery rate
GO:0033554	Cellular response to stress	9 of 1725	1.05	4.23e-06
GO:0051052	Regulation of DNA metabolic process	6 of 360	1.56	2.11e-05
GO:0000019	Regulation of mitotic recombination	3 of 6	3.04	2.41e-05
GO:0045935	Positive regulation of nucleobase-containing compound me...	8 of 1927	0.96	0.00024
GO:0032392	DNA geometric change	4 of 114	1.88	0.00040
GO:0071103	DNA conformation change	5 of 328	1.52	0.00040
GO:0090398	Cellular senescence	3 of 33	2.3	0.00064
GO:0006979	Response to oxidative stress	5 of 393	1.44	0.00064
GO:1901990	Regulation of mitotic cell cycle phase transition	5 of 416	1.42	0.00064
GO:0001932	Regulation of protein phosphorylation	7 of 1459	1.02	0.00064
GO:1900117	Regulation of execution phase of apoptosis	3 of 41	2.2	0.00085
GO:0046825	Regulation of protein export from nucleus	3 of 42	2.19	0.00085
GO:1901796	Regulation of signal transduction by p53 class mediator	4 of 182	1.68	0.00085
GO:0051054	Positive regulation of DNA metabolic process	4 of 200	1.64	0.0010
GO:0006281	DNA repair	5 of 522	1.32	0.0011
GO:0051338	Regulation of transferase activity	6 of 1036	1.1	0.0011
GO:1901991	Negative regulation of mitotic cell cycle phase transition	4 of 219	1.6	0.0012
GO:0051276	Chromosome organization	6 of 1066	1.09	0.0012
GO:0031860	Telomeric 3 overhang formation	2 of 4	3.04	0.0014
GO:0034599	Cellular response to oxidative stress	4 of 244	1.55	0.0014
GO:0051130	Positive regulation of cellular component organization	6 of 1209	1.03	0.0018
GO:0007568	Aging	4 of 274	1.5	0.0020
GO:0031401	Positive regulation of protein modification process	6 of 1252	1.02	0.0020
GO:0051348	Negative regulation of transferase activity	4 of 294	1.47	0.0023
GO:0010628	Positive regulation of gene expression	7 of 2337	0.81	0.0032
GO:0010604	Positive regulation of macromolecule metabolic process	8 of 3600	0.68	0.0032
GO:0051128	Regulation of cellular component organization	7 of 2402	0.8	0.0036
GO:0051352	Negative regulation of ligase activity	2 of 11	2.6	0.0039
GO:1903800	Positive regulation of production of miRNAs involved in gen...	2 of 11	2.6	0.0039
GO:0032508	DNA duplex unwinding	3 of 106	1.79	0.0039
GO:2000134	Negative regulation of G1/S transition of mitotic cell cycle	3 of 107	1.78	0.0039
GO:0033044	Regulation of chromosome organization	4 of 359	1.38	0.0039
GO:1902510	Regulation of apoptotic DNA fragmentation	2 of 12	2.56	0.0042
GO:0090399	Replicative senescence	2 of 13	2.52	0.0046
GO:0045893	Positive regulation of transcription, DNA-templated	6 of 1587	0.91	0.0046
GO:1903799	Negative regulation of production of miRNAs involved in ge...	2 of 14	2.49	0.0050
GO:0090343	Positive regulation of cell aging	2 of 15	2.46	0.0051

Since all nine SNPs were located in genes that are centered around the DNA damage repair processes, it is to be expected that they are interconnected (we attach the list of interactions of pairs of our SNPs).

node1	node2	node1 accession	node2 accession	node1 annotation	node2 annotation	score
CDKN2A	CDKN2B	ENSP00000418915	ENSP00000276925	Cyclin-dependent kinase inhibitor 2A; Act	Cyclin-dependent kinase 4 inhibitor B; Interacts stro	0.951
CDKN2A	IL6	ENSP00000418915	ENSP00000385675	Cyclin-dependent kinase inhibitor 2A; Act	Interleukin-6; Cytokine with a wide variety of biolo	0.621
CDKN2A	MRE11	ENSP00000418915	ENSP00000325863	Cyclin-dependent kinase inhibitor 2A; Act	Double-strand break repair protein MRE11; Compor	0.448
CDKN2A	TP53	ENSP00000418915	ENSP00000269305	Cyclin-dependent kinase inhibitor 2A; Act	Cellular tumor antigen p53; Acts as a tumor suppres	0.999
CDKN2B	CDKN2A	ENSP00000276925	ENSP00000418915	Cyclin-dependent kinase 4 inhibitor B; Int	Cyclin-dependent kinase inhibitor 2A; Acts as a neg	0.951
CDKN2B	TP53	ENSP00000276925	ENSP00000269305	Cyclin-dependent kinase 4 inhibitor B; Int	Cellular tumor antigen p53; Acts as a tumor suppres	0.86
ERCC2	MRE11	ENSP00000375809	ENSP00000325863	General transcription and DNA repair fact	Double-strand break repair protein MRE11; Compor	0.619
ERCC2	TP53	ENSP00000375809	ENSP00000269305	General transcription and DNA repair fact	Cellular tumor antigen p53; Acts as a tumor suppres	0.804
IL6	CDKN2A	ENSP00000385675	ENSP00000418915	Interleukin-6; Cytokine with a wide variet	Cyclin-dependent kinase inhibitor 2A; Acts as a neg	0.621
IL6	TP53	ENSP00000385675	ENSP00000269305	Interleukin-6; Cytokine with a wide variet	Cellular tumor antigen p53; Acts as a tumor suppres	0.777
MRE11	CDKN2A	ENSP00000325863	ENSP00000418915	Double-strand break repair protein MRE1	Cyclin-dependent kinase inhibitor 2A; Acts as a neg	0.448
MRE11	ERCC2	ENSP00000325863	ENSP00000375809	Double-strand break repair protein MRE1	General transcription and DNA repair factor IIH heli	0.619
MRE11	RAD50	ENSP00000325863	ENSP00000368100	Double-strand break repair protein MRE1	DNA repair protein RAD50; Component of the MRN	0.999
MRE11	TP53	ENSP00000325863	ENSP00000269305	Double-strand break repair protein MRE1	Cellular tumor antigen p53; Acts as a tumor suppres	0.905
PARK7	TP53	ENSP00000340278	ENSP00000269305	Protein/nucleic acid deglycase DJ-1; Prote	Cellular tumor antigen p53; Acts as a tumor suppres	0.814
PARK7	TXNRD1	ENSP00000340278	ENSP00000434516	Protein/nucleic acid deglycase DJ-1; Prote	Thioredoxin reductase 1, cytoplasmic; Isoform 1 ma	0.493
RAD50	MRE11	ENSP00000368100	ENSP00000325863	DNA repair protein RAD50; Component of	Double-strand break repair protein MRE11; Compor	0.999
RAD50	TP53	ENSP00000368100	ENSP00000269305	DNA repair protein RAD50; Component of	Cellular tumor antigen p53; Acts as a tumor suppres	0.709
TP53	CDKN2A	ENSP00000269305	ENSP00000418915	Cellular tumor antigen p53; Acts as a tumo	Cyclin-dependent kinase inhibitor 2A; Acts as a neg	0.999
TP53	CDKN2B	ENSP00000269305	ENSP00000276925	Cellular tumor antigen p53; Acts as a tumo	Cyclin-dependent kinase 4 inhibitor B; Interacts stro	0.86
TP53	ERCC2	ENSP00000269305	ENSP00000375809	Cellular tumor antigen p53; Acts as a tumo	General transcription and DNA repair factor IIH heli	0.804
TP53	IL6	ENSP00000269305	ENSP00000385675	Cellular tumor antigen p53; Acts as a tumo	Interleukin-6; Cytokine with a wide variety of biolo	0.777
TP53	MRE11	ENSP00000269305	ENSP00000325863	Cellular tumor antigen p53; Acts as a tumo	Double-strand break repair protein MRE11; Compor	0.905
TP53	PARK7	ENSP00000269305	ENSP00000340278	Cellular tumor antigen p53; Acts as a tumo	Protein/nucleic acid deglycase DJ-1; Protein and nu	0.814
TP53	RAD50	ENSP00000269305	ENSP00000368100	Cellular tumor antigen p53; Acts as a tumo	DNA repair protein RAD50; Component of the MRN	0.709
TP53	TXNRD1	ENSP00000269305	ENSP00000434516	Cellular tumor antigen p53; Acts as a tumo	Thioredoxin reductase 1, cytoplasmic; Isoform 1 ma	0.609
TXNRD1	PARK7	ENSP00000434516	ENSP00000340278	Thioredoxin reductase 1, cytoplasmic; Iso	Protein/nucleic acid deglycase DJ-1; Protein and nu	0.493
TXNRD1	TP53	ENSP00000434516	ENSP00000269305	Thioredoxin reductase 1, cytoplasmic; Iso	Cellular tumor antigen p53; Acts as a tumor suppres	0.609

The only SNP not related with others is the one in the TERC gene, but since it encodes an RNA component of the telomerase enzyme, it is therefore not listed in protein databases.
